# A New Flavone *C*-Glycoside from *Clematis rehderiana*

**DOI:** 10.3390/molecules15020672

**Published:** 2010-01-29

**Authors:** Zhi-Zhi Du, Xian-Wen Yang, Hao Han, Xiang-Hai Cai, Xiao-Dong Luo

**Affiliations:** 1 State Key Laboratory of Phytochemistry and Plant Resources of West China, Kunming Institute of Botany, Chinese Academy of Sciences, Kunming 650204, China; E-Mail: duzhizhi@mail.kib.ac.cn (Z.-Z.D.); 2 Key Laboratory of Marine Bioresources Sustainable Utilization, South China Sea Institute of Oceanology, Chinese Academy of Sciences, Guangzhou 510301, China; E-Mail: yangxw76@163.com (X.-W.Y.)

**Keywords:** *Clematis rehderiana*, flavonoide glycosides, antioxidant

## Abstract

A new flavone *C*-glycoside, isovitexin 6″-*O*-*E*-*p*-coumarate (**1**) and two known flavonoid glycosides—quercetin 3-*O*-β-D-glucuronopyranoside (**2**) and isoorientin (**3**)—were isolated from an ethanol extract of aerial parts of *Clematis rehderiana*. Their structures were determined by spectroscopic methods. The antioxidant effects of the two flavone *C*-glycosides were evaluated by both the MTT and DPPH assays. Compound **1** showed potent activities against H_2_O_2_-induced impairment in PC12 cells within the concentration range tested, whereas compound **3** scavenged DPPH radical strongly, with an IC_50_ value of 13.5 μM.

## Introduction

The genus *Clematis* (Ranunculaceae), which comprises about 300 species, is widespread throughout the World. About 147 species (93 endemic ones) are distributed in China, and 56 of these are distributed in Yunnan province [1,2]. It is reported that 77 *Clematis* species have been used in traditional Chinese medicine, of which 32 are found in Yunnan province [3]. The genus *Clematis* has many different pharmacological effects such as antibacterial, anti-inflammatory, antitumor, analgesic and diuretic functions [4]. Reports on the chemical components of genus *Clematis* have been scarce up to now and mainly refer to triterpenoid saponins [4,5,6]. In order to provide some knowledge for better usage of the *Clematis* resources, we have investigated the chemical constituents and antioxidant activity of *Clematis rehderiana* from Yunnan. 

*C. rehderiana* (English common name: cowslip scented clematis) is distributed in northwest Yunnan and used by local Tibetan people as a diuretic for eliminating dampness and cure indigestion, lumps in the abdomen and skin ulcers [7]. To the best of our knowledge, there are no reports about the chemical constituents of the species. We report here the isolation and characterization of one new and two known flavonoid glycosides from this plant, as well as antioxidant assay results for two of these compounds. 

## Results and Discussion

### Structure Elucidation

The extract of *C. rehderiana* obtained with 90% EtOH was then successively partitioned between H_2_O and EtOAc, followed by H_2_O and *n*-butanol. The *n*-butanol soluble fraction was separated by different chromatographic procedures to afford one new *C*-flavone glycoside **1** and two known flavonoid glycosides **2 **and **3**. Their structures were determined by examination of their spectral data and comparison of the data with reported literature values. 

**Figure 1:**
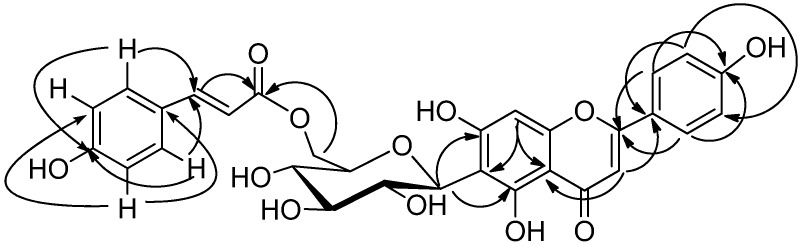
Key HMBC interactions of compound **1**.

**Figure 2:**
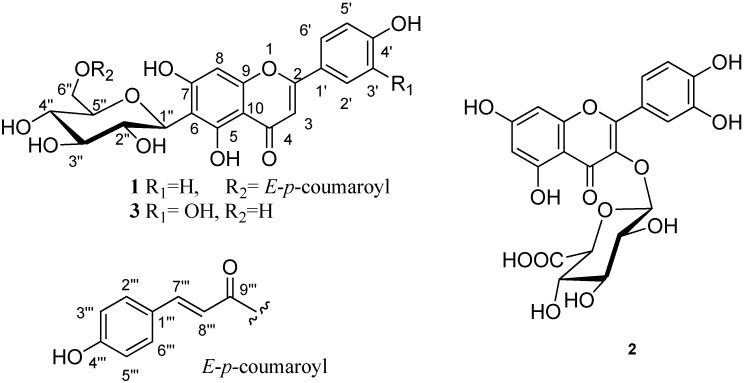
Structures of compounds **1**–**3**.

Compound **1** was obtained as a yellow powder. The molecular formula C_30_H_26_O_12_ was determined based on the high resolution FABMS data (*m*/*z* 577.1352 [M−H]‾, calcd: 577.1346). It gave a positive green coloration with 1% FeCl_3_ reagent suggesting it was a flavonoid. The ^1^H-NMR spectrum of **1 **(Table 1) analyzed with the aid of HSQC and HMBC, showed two hydroxyl proton signals at δ_H_ 13.64 and δ_H_ 9.88; two one-proton singles at δ_H_ 6.54 and δ_H_ 6.63, attributed to H-8 and H-3, respectively; and two doublets at δ_H_ 7.91 (2H, d, *J* = 8.8 Hz, H-2′, 6′) and δ_H_ 6.98 (2H, d, *J* = 8.8 Hz, H-3′, 5′), suggesting that **1** was a 5,7,4′-trihydroxyflavone derivative. The ^1^H-NMR also showed sugar proton signals at δ_H_ 3.54 (2H, m), 3.70 (1H, m), 4.12 (1H, m), 4.34 (1H, dd, *J* = 6.0, 12 Hz), 4.56 (1H, dd, 1.52, 12.0 Hz), 4.92 (1H, d, *J* = 9.8 Hz) attributed to H-4″ and 5″; 3″; 2″; 6″; 6″ and the anomeric proton H-1″. The position of attachment of the sugar moiety to the flavonol skeleton was determined by HMBC experiments which showed long range coupling between the anomeric H-1″ (δ 4.92) and the C-5 at δ 161.7 and C-7 at δ 164.1. The ^1^H- and ^13^C-NMR spectroscopic data of the aglycone and sugar moieties of **1** were similar to those of isovitexin [8], except for the presence of nine additional carbon signals. These nine carbon signals are compatible with an *E*-*p*-coumaroyl moiety [9]. Two doublets at δ_H_ 6.85 (*J* = 8.6 Hz) and δ_H_ 7.53 (*J* = 8.6 Hz) were attributed to H-3'''/H-5''' and H-2'''/H-6'''of the *E*-*p*-coumaroyl moiety, respectively. Two other doublets (*J* = 15.9 Hz) at δ_H_ 6.37 and δ_H_ 7.62 were assigned to H-8''' and H-7''' of the *p*-coumaroyl moiety with *E*-configuration, respectively. The acylation of C-6″ with a *p*-coumaroyl unit was deduced from the downfield shift of CH_2_-6″ diastereomeric protons (~ + 1 ppm) and its ^13^C-resonance at 64.7 (+ 4 ppm). This evidence was confirmed by the HMBC correlation between δ_H_ 4.34, 4.56 (H-6″) and C-9''' at δ 167.5 Figure 1). Based on the spectral data, the structure of **1** was characterized as the new natural product isovitexin 6″-*O*-*E*-*p*-coumarate (Figure 2).

Compound **2** was isolated as a yellow powder and had a molecular formula of C_21_H_18_O_13_, as determined by the high resolution FABMS (*m*/*z* 477.0669 [M−H]‾). The ^1^H-NMR showed two doublets at δ_H_ 6.83 (1H, d, *J* = 8.3 Hz, H-2′) and δ_H_ 7.42 (1H, br d, *J* = 8.2 Hz, H-6′) and one singlet at δ_H_ 8.05 (H-5′), suggesting (*ortho*)-hydroxylation of the B-ring, whereas 5,7-dihydroxylation of A-ring was duduced from two *meta*-coupled doublets that appeared as br-singlets at δ_H_ 6.39 (H-8) and δ_H_ 6.19 (H-6). The presence of a glucuronide moiety was suggested by the signals at δ_H_ 3.11–3.25 (3H, m, H-2″, 3″, 4″), δ_H_ 5.32 (H-1″) and a set of carbon signals at δ_C _102.4 (C-1″), 74.1 (C-2″), 76.4 (C-3″), 71.7 (C-4″), 74.7 (C-5″) and 171.7 (C-6″). The assignment of ^1^H- and ^13^C-NMR resonances for the glucuronide moiety were decided by HMBC and H–H COSY correlations, as well as comparison with the reported literature data [10]. The position of attachment of glucuronide moiety to the flavonol skeleton was determined by HMBC experiments which showed long range correlation (^3^J-coupling) between the anomeric proton H-1″ (δ 5.32) and the C-3 (δ 133.8). It was further confirmed by the absence of the characteristic H-3 proton singlet at around δ_H_ 6.7 associated with the C-3 in the C-ring [10]. Therefore, compound **2** was identified as quercetin 3-*O*-β-D-glucuronopyranoside (miquelianin; Figure 2). Finally, compound **3** was identified as isoorientin (Figure 2) by its spectral data (Table 1) and comparison of this data with the reported literature values [8].

**Table 1:** ^1^H- and ^13^C-NMR data of compounds **1 **and **3** in DMSO (δ in ppm, *J* in Hz).*

Position	Compound 1		Compound 3
	δ_C_	δ_H_	δ_C_
2	164.9		163.7
3	103.7	6.63 (s)	102.8
4	183.1		181.9
5	161.7		160.7
6	109.0		108.9
7	164.1		163.3
8	95.0	6.54 (s)	93.5
9	157.7		156.2
10	104.8		103.4
1′	122.5		121.5
2′	129.0	7.91 (d, 8.8)	113.3
3′	116.8	6.98 (d, 8.8)	145.8
4′	162.4		149.8
5′	116.8	6.98 (d, 8.8)	116.1
6′	129.0	7.91 (d, 8.8)	119.0
1″	74.9	4.92 (d, 9.8)	73.1
2″	72.3	4.12 (m)	70.2
3″	79.4	3.70 (m)	79.0
4″	71.3	3.54 (m)	70.7
5″	79.7	3.54 (m)	81.7
6″	64.7	4.56 (dd, 1.52, 12.0)	61.6
		4.34 (dd, 6.0, 12.0)	
1'''	126.3		
2'''	130.9	7.53 (d, 8.6)	
3'''	116.6	6.85 (d, 8.6)	
4'''	161.1		
5'''	116.6	6.85 (d, 8.6)	
6'''	130.9	7.53 (d, 8.6)	
7'''	145.7	7.62 (d, 15.9)	
8'''	114.9	6.37 (d, 15.9)	
9'''	167.5		

^*^^ 1^H- and ^13^C-NMR spectra were obtained at 500 and 125 MHz, respectively

### Biological Activity

Since phenolics are characterized by antioxidant activities [11,12,13,14], two isolates were subjected to antioxidant activity experiments using both the MTT and DPPH assays (Table 2 and Table 3). Compound **1** showed potent activity against H_2_O_2_-induced impairment in PC12 cells within the concentration range tested (0.4 to 50 μM), whereas isoorientin (**3**) scavenged DPPH radical strongly, with an IC_50_ value of 13.5 μM. This indicated that compound **1** might be an indirectly acting antioxidant, while **3** might be a directly acting antioxidant.

**Table 2:** Antioxidant effects of compounds **1** and **3** against H_2_O_2_-induced impairment in PC12 cells.

Groups	Concentration (μM)	Viability (%)
Control		100
Model *^a^*		40.4 ± 4.6 ***
Edaravone *^b^*	10.0	35.3 ± 3.2
	2.0	43.2 ± 4.0
	0.4	46.1 ± 2.0 *
	0.08	44.3 ± 2.5
Compound **1**	50.0	64.9 ± 9.8 ***
	10.0	53.7 ± 7.0 **
	2.0	46.1 ± 6.6
	0.4	47.6 ± 10.2
Compound **3**	50.0	43.7 ± 4.8
	10.0	46.7 ± 5.2
	2.0	44.9 ± 6.8
	0.4	44.5 ± 6.2

*^a^* Negative control; *^b^* Positive control; *n* = 5; * *p* < 0.05; ** *p* < 0.01; *** *p* < 0.001 *vs*. model.

**Table 3:** Radical scavenging activities of compound **1** and **3** against DPPH.

Compounds	IC_50_ ( μM )
Edaravone *^a^*	26.0
**1**	> 100
**3**	13.5

*^a^* Positive control.

## Experimental

### General

Optical rotations were measured with a JASCO DIP-370 digital polarimeter in MeOH solutions. IR spectra were measured on a Bio-Rad FTS-135 infrared spectrometer with KBr pellets. UV spectra were obtained with a Shimadzu UV-2401PC spectrometer. Mass spectra were measured on a VG Auto Spec-3000 spectrometer. NMR spectra were recorded on a DRX-500 NMR spectrometer with TMS as internal standard. Silica gel (200-300 mesh) for column chromatography and precoated TLC plates (Si gel G) were purchased from the Qingdao Marine Chemical Factory (Qingdao, P. R. China). Reversed-phase C_18_ silica gel for column chromatography were obtained from Merck. Sephadex LH-20 for column chromatography was purchased from Amersham Biosciences. 

### Plant material

Aerial parts of *Clematis rehderiana* was collected from Lijian in Yunnan Province, China, in August 2005. A voucher specimen (KUN0816301) is stored at the herbarium of Kunming Institute of Botany, Chinese Academy of Sciences.

### Extraction and isolation

The air-dried aerial parts of *C. rehderiana* (3 kg) were ground and extracted four times with 90% EtOH (8 L each time) at room temperature. After removal of the solvent, the residue was suspended in water (2 L) and then extracted successively with petroleum ether (PE), EtOAc and *n*-butanol (4 × 2L each). The *n*-butanol extract was subjected to column chromatography (CC) over macroporous resin D101 eluting with EtOH-H_2_O (0%–95%) and afforded five fractions Fr. I–V. Fr. III (18.1 g) was subjected to CC over silica gel (100 g) and eluted with CHCl_3_/MeOH 20:1→ 9:1→ 4:1 → 7:3 and EtOH to give 10 subfractions (Fr. III1 – Fr. III10). Fr. III5 was further isolated by repeated vacuum liquid chromatography (VLC) over RP-18 and eluted with MeOH-H_2_O (0% to 50%) to afford compounds **2** (4 mg) and **3** (50 mg). Fr. IV (3.9 g) was subjected to CC over silica gel (60 g) and eluted with CHCl_3_/MeOH 20:1→ 9:1→ 7:3 to give 14 subfractions (Fr. IV1 – Fr. III14). Fr. IV8 was further fractionated by repeated CC over SephadexLH-20 and eluted with MeOH-CHCl_3_ and MeOH to afford compound **1 **(12 mg).

*Compound*** 1**: 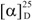 = +32.9 (*c* 0.76, MeOH); IR (KBr): 3,420, 2,920, 1,720 cm^-1^; ^1^H- and ^13^C-NMR see Table 1. HRFABMS (neg): *m*/*z* 577.1352 [M-H]^−^, calcd: 577.1346.

*Compound*** 2**: 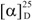 = −8.6 (*c* 0.58, MeOH); UV (MeOH) λ_max_ (log ε) 404, 270, 210 nm; IR (KBr): 3,408, 2,922, 1,653, 1,604; HRFABMS (neg): *m*/*z* 477.0669 [M-H]^−^, calcd: 477.0669; ^1^H-NMR (DMSO-*d_6_*) δ: 12.35 (1H, br s 5-OH), 8.05 (1H, s, H-5′), 7.42 (1H, br d, *J* = 8.2 Hz, H-6′), 6.83 (1H, d, *J =* 8.3 Hz, H-2′), 6.39 (1H, br s, H-8), 6.19 (1H, br s, H-6), 5.32 (1H, d, *J =* 7.0 Hz, H-1″), 3.39 1H, m, H-5″), 3.11-3.25 (3H, m, H-2″, 3″, 4″); ^13^C-NMR (DMSO-*d_6_*) δ: 157.2 (C-2), 133.8 (C-3), 177.4 (C-4), 161.0 (C-5), 98.9 (C-6), 164.5 (C-7), 93.7 (C-8), 156.4 (C-9), 103.7 (C-10), 120.6 (C-1′), 115.3 (C-2′), 144.8 (C-3′), 148.5 (C-4′), 117.5 (C-5′), 120.9 (C-6′), 102.4 (C-1″), 74.1 (C-2″), 76.4 (C-3″), 71.7 (C-4″), 74.7 (C-5″), 171.7 (C-6″).

### Antioxidant assays

The antioxidant assay against H_2_O_2_-induced impairment in PC12 cells was conducted according to the reported protocol [14]. Briefly, PC12 cells were seeded into 96-well plates in RPMI 1640 medium with 10% characterized Newborn Bovine Serum. Twenty-four hours later, different concentrations of compounds **1** and **3** together with freshly prepared H_2_O_2 _were added and incubation continued for 1 hour. Then MTT (3-(4,5-dimethylthiazol-2-yl)-2,5-diphenyltetrazolium bromide) solution was added and the incubation continued for 4 hours. Finally, solution (100 μL) containing 5% *iso*-butanol, 10% SDS (Sigma) and 0.004% HCl was added. The mixtures were kept overnight and the index of cell viability (% of control) was calculated by measuring the optical density of the color produced by MTT dye reduction with a microplate reader at 570 nm.

DPPH radical-scavenging activity assays were performed according to our previously reported procedures [11]. Each compound (100 μL) at five different concentrations was added to DPPH solution (100 μL). The absorbance was measured with a microplate reader at 517 nm after 30 min of reaction at 37 °C. IC_50_ values denote the concentration of sample required to scavenge 50% DPPH free radicals.

## Conclusions

A new flavone *C*-glycoside, isovitexin 6″-*O*-*E*-*p*-coumarate (**1**) and two known flavonoid glycosides—quercetin 3-*O*-β-D-glucuronopyranoside (**2**) and isoorientin (**3**)—were isolated from an ethanol extract of aerial parts of *C. rehderiana*. The antioxidant effects experiments of these compounds by both the MTT and DPPH assays suggested that flavone glycosides were major constituents with antioxidant activities in this plant. 
